# Feasibility of Implementing a Tai Chi Program in an Assisted Living Facility: Reducing Fall Risks and Improving Quality of Life

**DOI:** 10.3390/jcm10061277

**Published:** 2021-03-19

**Authors:** Yingying Chen, Deborah Ringdahl, Rachel Trelstad-Porter, Olga V. Gurvich

**Affiliations:** 1 Edson College of Nursing and Health Innovation, Arizona State University, Phoenix, AZ 85004, USA; 2 School of Nursing, University of Minnesota, Minneapolis, MN 55455, USA; ringd001@umn.edu (D.R.); ogurvich@umn.edu (O.V.G.); 3 Senior Care Communities, Inc, St. Paul, MN 55103, USA; RTrelstadPorter@woodburyseniorliving.com

**Keywords:** postural balance, falls, functional status, movement, Tai Chi Chuan, Tai-ji, older adults, movement, aged

## Abstract

One in four American older adults fall every year, resulting in injuries, death, and significant financial burden. Although fall etiology is multifactorial, the medical problems and aging factors that lead to unsteady gait and imbalance represent one of the major fall risks among older adults. A growing number of research studies support the health benefits of regular Tai Chi (TC) practice including improved physical, cognitive, and psychological function. The purpose of this quality improvement project was to assess the feasibility of establishing a 12-week (45 min per session) Tai Chi (TC) program (Sun Style Tai Chi) in a 75 bed assisted living facility as well as to evaluate the potential of the TC program to improve the fear of falling and functional mobility (as proxy for fall risk) and quality of life (QoL). A nurse who was a certified TC instructor taught the program. Twenty-three participants, 96% female and 96% white, mean (SD) age 83 (±7) years, attended one or more TC classes. Class attendance, self-reported questionnaires (e.g., fear of falling, QoL), and objective measure Timed Up and Go (TUG) were used to collect data. Nine participants (39%) completed 9 out of 12 sessions. Eleven participants (48%) completed both pre- and post-intervention measurements and twelve (52%) provided feedback on a post-intervention satisfaction survey. Participants showed 20% improvement in fear of falling (mean relative change) and 21% decrease (mean relative change) in TUG test (*p* = 0.001) with no clinically important changes in QoL. This quality improvement project suggested that TC is a feasible exercise that might have the potential to reduce risk of falls in older adults, and the program was well accepted with no serious or other adverse events reported. Further research studies are needed to examine the potential effects of TC programs with an appropriately powered RCT and longer intervention period.

## 1. Introduction

### 1.1. Background

More than one in four adults aged 65 and over falls every year in the United States, resulting in 3 million emergency visits and over 800,000 hospitalizations. These falls contribute to an increasing death rate each year for older adults [1]. Falls lead to injuries and disabilities that reduce older adults’ quality of life and threaten their ability to live independently [1]. Falls also contribute to fear of falling, which may in turn reduce daily activities and social interaction. This lack of activity and interaction can result in decreased mobility, social isolation, poor muscle strength, and diminished balance [2,3]. Furthermore, the substantial economic costs associated with falls impose a serious and growing financial burden at both the individual and societal level. In 2015, direct fatal and non-fatal fall related health care costs in the United States exceeded $637 million and $31 billion, respectively [4]. Thus, there is an urgent need to reduce the risk of falls among the elderly with the goal of preventing injury, reducing healthcare costs, and improving quality of life in this population.

Although fall etiology is multifactorial, the medical problems and aging factors that lead to unsteady gait and imbalance represent some of the major fall risks among older adults [5]. Experts recommend that exercise targeting balance, strength, and gait training be offered to community-dwelling seniors [6]. The Centers for Disease Control and Prevention (CDC) identified 14 evidence-based exercise interventions, three of which are Tai Chi-based, for fall prevention targeting community-dwelling older adults [7]. A systematic review spanning 20 years suggested that Tai Chi has a positive impact on balance and mobility for older adults [8].

### 1.2. Tai Chi

Tai Chi, originating in China and derived from Chinese martial arts, has been practiced for hundreds of years in China to enhance people’s wellbeing. In the past few decades, Tai Chi (TC) has gained popularity in Western countries. TC is characterized by slow, gentle, continuous, and mindful movements accompanied by synchronized breathing aiming to improve physical and mental health [8]. Experts suggest that TC is an effective and safe practice for older adults, even those with compromised mobility [9,10]. Systematic literature reviews [8,9] also suggested TC is a safe and cost- effective practice.

TC has been linked to a variety of health benefits including physical and mental health as well as improved quality of life [11,12]. In particular, strong scientific evidence suggests that TC has promising effects on fall reduction among older adults [13,14]. The results are consistent with the findings in previous systematic reviews [15,16] and in accordance with the most recent meta-analysis indicating TC programs could reduce the number of falls and injury-related falls within a year by 43% and 50%, respectively [17]. Recent empirical studies indicate Tai Chi has equal or greater health benefits in comparison to aerobic exercise [12], and in one study Tai Chi outperformed conventional exercise in fall prevention among community seniors [18]. Compared to those who did not practice Tai Chi, older adults who practiced Tai Chi showed better balance, strength, mobility, and confidence (reduced fear of falling) [14]. Finally, an evaluation study that implemented a 12-week evidence-based TC program found that the program was well-received by older adults, has potential to reduce fall risks, and can be implemented in community settings [19]. Taking these studies into consideration, TC was adopted as the intervention strategy to reduce falls and improve quality of life (QoL) in older adults in the quality improvement program described in this paper.

### 1.3. Social Cognitive Theory

The Social Cognitive Theory (SCT) [20,21] was used as the theoretical framework to guide implementation of this evidenced-based TC program. SCT has been widely used to guide behavioral changes and health promotion among various populations. This theory posits that three factors influence each other bidirectionally; these factors can be personal (e.g., age, mobility, and self-efficacy), behavioral, and environmental (e.g., physical environment, and psychosocial environment). According to SCT, participants’ behavior (engagement in TC classes) may be predicted by the enjoyment and satisfaction derived from the class as well as the supportive environment provided by the class. 

### 1.4. Purpose Statement

Although mounting empirical evidence suggests TC is an effective approach in reducing fall risk and public health authorities recommend and endorse it, older adults in assisted living facilities have limited access to TC programs. There are many barriers to translating and disseminating TC research findings into community practice [22,23]. One of the major barriers is that many studies have been conducted in well-controlled environments that do not fit well with real world situations [22]. In response, this quality improvement program attempted to fill the literature gap by implementing a TC program at an assisted living facility and assessing its feasibility and safety in this population and setting. The secondary exploratory purpose of this project was to evaluate the potential of a TC program to improve fear of falling and functional mobility, which in turn might reduce the risk of falls and improve QoL. Based on the literature review and previous studies, it was hypothesized that: (1) it would be feasible, safe, and acceptable to implement the TC program at an assisted living facility; and (2) the TC program would demonstrate a preliminary potential to improve fear of falling and functional mobility and to improve QoL.

## 2. Methods

### 2.1. Ethical Consideration 

This project was reviewed for Human Subject Protection using the University of Minnesota online Institutional Review Board (IRB) determination tool and met the criteria for Quality Assurance (QA)/Quality Improvement (QI). Therefore, no additional IRB approval was required for this project.

### 2.2. Setting 

This quality improvement project took place at a 75-bed assisted living facility, which was part of a senior care center located in east suburban Minnesota. A comprehensive needs assessment was conducted by interviewing facility leaders, staff, and residents, revealing significant interest in developing and implementing a Tai Chi program. This senior care center had no prior Tai Chi program, but there was an existing exercise program for assisted living residents that consisted of weightlifting exercise, chair yoga, ball exercise, and drumming. These residents had access to a daily 30 min exercise program, with daily attendance fluctuating between 5 and 12 participants. This exercise program had been offered for the past 5 years, and the organization had expressed a growing interest in expanding the program to include a weekly evidence-based Tai Chi program to enhance the health and wellbeing of their residents. 

### 2.3. Participants 

The inclusion criteria for participation in this study were: (1) ≥55 years old (the facility is open to qualified adults who are ≥55 years old and the program was intended to be inclusive to all residents in the facility) (2) did not have cognitive impairment or mental disabilities that might limit the practice of TC; and (3) possessed basic English communication capability. The participants were not excluded if they needed an assistive device (such as a cane, walker, or wheelchair) for locomotion. Participants excluded themselves if their medical providers discouraged them from participating in TC. The exercise programs were open to all residents interested in attending. All participants were residents living independently or with some assistance in the 75-bed assisted living building. Program recruitment was initiated with a 45-min educational presentation that was available to residents and staff. Informational flyers about the educational presentation were posted in areas visible to residents and staff, and table flyers were placed on the dining tables for residents to view during mealtime. The presentation was offered one week prior to the first TC class and included: (1) an introduction to the quality improvement project; (2) TC philosophy and benefits; and (3) TC experiential practice. Twenty residents, the Community Life Director, and the Director of Integrative Health and Wellness attended the presentation. 

### 2.4. Design 

The project utilized a quasi-experimental, one group pre-test/post-test design. The TC program was available to all residents who were interested in attending the class and were able to be physically present in the activity room where the class was held. 

### 2.5. TC Intervention

Weekly Tai Chi classes were held for 12 consecutive weeks from September to November 2017 in the assisted living facility’s activity room and taught by a certified TC instructor who was also a registered nurse and the project leader. The TC program was adapted from Tai Chi for Arthritis and Fall Prevention program, which was designed by Dr. Paul Lam to reduce pain, prevent falls, improve balance, and promote general wellbeing [24]. The program was based on Sun Style of Tai Chi that has a higher stance with less punching and kicking, making it suitable for older adults [25]. An earlier study [26] indicates the Tai Chi for Arthritis and Fall Prevention program significantly reduced the incidence of falls as well as the fall risks. Full endorsement for this program was received from the CDC, with the recommendation to use with community dwelling seniors for fall prevention. 

Each 45-min class was structured with the following activities: 15–20 min of TC warm up exercises, 5 min for water break, 15–20 min of TC practice, and a 5-min cool-down. To accommodate the residents’ mobility, the class started with seated movements, progressed to options of standing with or without chair assist, and then ended with seated relaxation movements. Synchronized breathing (breathing with the movements) and guided imagery (e.g., imaging feet rooting in the ground, imaging self as a steady mountain) were integrated into TC movements. The Community Life Director and the Director of Integrative Health and Wellness participated in all the classes in order to learn TC movements with the residents, support the program, and monitor the safety of the participants. During the very last 4 weeks, the two directors participated in co-teaching with the TC instructor, and received direct feedback after each class. At the end of the program implementation, a one-hour private training session was provided for the directors to support teaching confidence and TC program continuation. 

Written handouts with TC photos and descriptions of movements were provided to participants. Other supporting materials were also given to the facility to support staff education and program sustainability including written scripts of the teaching, the book of “Teaching Tai Chi Effectively”, an official DVD of the Tai Chi for Arthritis program created by Dr. Paul Lam, which demonstrated the movements that were taught in classes, and two Tai Chi music CDs. At the end of the program, photo books, depicting each TC movement in pictures, were created by the instructor in collaboration with the residents and staff. Class participants received a commemorative copy of the photo book. 

### 2.6. Measures 

Feasibility of this TC program, the primary aim of this project, was evaluated by recording class attendance and completion of a post-intervention satisfaction survey. At the end of the program, a 10-question satisfaction survey including 9 multiple choice questions and one open-ended question was distributed to the participants to evaluate their satisfaction and enjoyment of the program (see Appendix A). Safety was monitored by staff and instructor during every TC class. Safety in this project refers to being free of falls and injuries directly related to Tai Chi practice. Participants were instructed to report any adverse events including falls, injuries, pain, or any other discomfort resulting from Tai Chi practice.

Outcomes for the secondary aim, fear of falling, functional mobility, and QoL, were evaluated using three measures. Fear of falling and functional mobility were assessed by the short version of Falls Efficacy Scale-International (FES-I) [27], and the Timed Up and Go (TUG) test, respectively. The short FES-I assesses the fear of falling when performing seven common daily activities including dressing, bathing, getting in or out of a chair, going up or down stairs, reaching up, walking, and outing (going out to social events). The total score ranges from 7 to 28, with a higher total score indicating greater concern of falling. The reliability and validity have been tested and the short FES-I is very comparable to the original version of FES-I with Cronbach’s alpha 0.92 [27]. The TUG test is widely employed to assess older adults’ functional mobility and balance with acceptable validity and reliability [28]. Residents were asked to sit back in an armchair, stand up from the chair, walk a three-meter taped line on the floor at a normal speed, turn, walk back, and sit down. The time used to accomplish the sequence was recorded as the outcome. An older adult who takes 12 s or longer to complete the TUG test is considered at high risk for falling.

QoL was assessed by the brief Older People’s Quality of Life (OPQOL-brief) questionnaire, consisting of 13 questions and one single item that assessed the global QoL that was included in the total score calculation. This is a short version of the widely used QoL questionnaire named OPQOL-35. The OPQOL-brief questionnaire has demonstrated acceptable validity and high reliability with Cronbach’s alpha 0.88 [29]. Each of the 13 items was scored (1 = strongly agree to 5 = strongly disagree), the items were summed for a total OPQOL-Brief score, then the positive items were reversely coded (per published scoring procedure). Higher scores represented higher QoL with a plausible score range of 13–65 [29].

### 2.7. Data Collection

Attendance was recorded by the instructor and confirmed by the Community Life Director during the weekly class. Satisfaction surveys were distributed at the end of the program to evaluate participants’ program satisfaction. Safety was monitored by staff and instructor to record any falls or injuries related to Tai Chi class. Short FES-I, TUG test, and OPQOL-brief were collected prior to the intervention in early September, and again at the end of the 12-week intervention on 24 November 2017. To protect the privacy of the participants, each participant was given a code to label their surveys. Collected data was entered into a spreadsheet on a password-protected computer and assessed for accuracy and completeness by the instructor.

### 2.8. Data Analysis

Descriptive statistics and graphical representations were used to summarize and analyze all collected data. Tools were scored according to published scoring procedures. Class attendance and post-intervention satisfaction survey results were summarized qualitatively and quantitatively. Absolute and relative changes in fear of falling, functional mobility, and QoL from pre- to post-intervention were estimated. Differences in TUG times from pre- to post-intervention were examined using paired t-test. Two-sided type I error rate of 5% was used to assess statistical significance. SPSS software (IBM Corp. Released 2013. IBM SPSS Statistics for Windows, Version 22.0. Armonk, NY, USA: IBM Corp.) was used for all analyses.

## 3. Results

### 3.1. Descriptive Analysis

Twenty-three out of 75 (31%) residents in the assisted living facility attended the TC program. Participants’ ages ranged from 70 to 93, with an average age 83 (±7) years, 96% participants were female and 96% were white. Participants included those who could walk independently without assistive devices and those who required a wheelchair, walker, or cane for locomotion. During the first class, participants were asked why they came for the TC class and if they had any experience with TC. Many participants stated that they came because they were curious about what TC was and hoped it would help them regain their balance and relieve pain. Most of the participants had heard about TC but never practiced it, with the exception of one who practiced TC for a short time several years ago.

### 3.2. Feasibility and Acceptability of the Program

Nine (39%) residents, average age 83 (±7), attended at least 9 out of 12 classes with an average of 11 participants per class. Eleven (48%) participants provided both pre- and post-intervention data. The satisfaction survey also indicated that participants were satisfied with the program and would like to continue with the program. Some residents commented that this new type of exercise stimulated their learning and believed it was beneficial to their memory. Staff observed no falls or injuries occurred during the implementation period. There were no serious or other adverse events associated with practicing Tai Chi reported by residents.

At the end of the program, 12 (52%) residents completed the satisfaction survey, including two items assessing residents’ enjoyment in and benefits of the TC class on a 4-point Likert Scale (1 = Strongly Disagree, 4 = Strongly Agree). They reported that they enjoyed the TC practice, with 50% selecting Strongly Agree and 50% Agree. Similarly, all believed the TC practice was helpful in improving their health and wellbeing, with half selecting Strongly Agree and the other half Agree. In addition, all of them reported (100%) that they would recommend the TC program to their friends and neighbors. They all agreed to continue the TC program. A total of 83% of the participants listed learning TC movements as their favorite part of the class, whereas scheduling conflicts and illnesses were mentioned as the major barriers for participating in the TC practice. A few participants consulted their primary care providers about TC practice and their confidence of making the right choice by practicing TC was boosted when affirmation of the positive benefits of TC were reinforced by their physicians. Another participant decided to stop practicing TC after her physician advised her to discontinue due to unknown medical reasons.

The two directors believed attending the classes, co-teaching, and private training sessions were very helpful in promoting greater confidence in teaching. A great interest was expressed in continuing the program after implementation period both from the residents and the leadership team.

### 3.3. Differences in Outcomes

**Figure 1:**
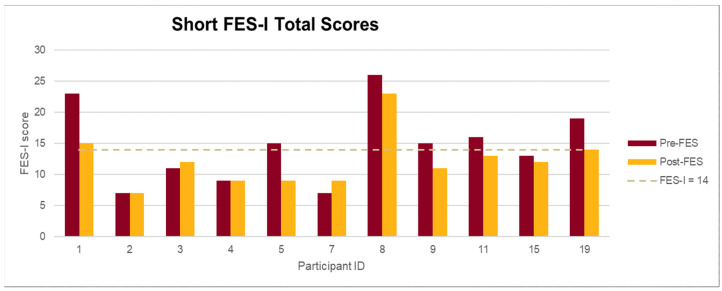
Falls Efficacy Scale-International (FES-I) score indicates level of concern of falling: Low: 7–8; Moderate: 9–13; High: 14–28. *n* = 11. FES-I plausible range 7–28, higher scores indicate higher concern.

Participants’ mean short FES-I score decreased from 15 at pre-test to 12 post-test, corresponding to a mean relative decrease of 20% in the fear of falling score. Notably, 7 out of 11 (64%) participants had lower FES-I scores at post-intervention as compared to pre-test (see Figure 1).

**Figure 2:**
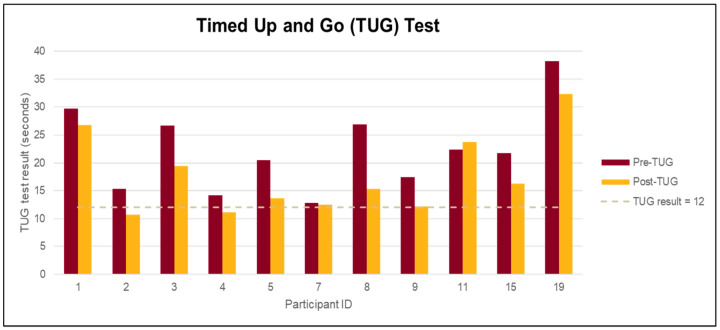
Timed Up and Go (TUG) ≥ 12 s indicates high risk for falling; *n* = 11. Longer times indicate higher risk for falling.

A mean decrease of 5 s (relative change of 21%) in mean TUG time (95% CI: (−2.4, −7.1), *p* = 0.001) from pre- to post-intervention was observed. Participants’ mean TUG time decreased from 22.4 at pre-test to 17.6 post-test. Ten out of 11 (90%) participants had lower mean TUG times at post-intervention as compared to pre-test (see Figure 2).

No clinically important changes in QoL (*p* > 0.05) as measured by OPQOL-brief score were observed (see Table 1). Global QoL showed some improvement from the pre-intervention to post-intervention (See Table 2).

## 4. Discussion

This project demonstrated that establishing a TC program at an assisted living facility is feasible. Although studies show that TC programs often had a high dropout rate [8], the TC program in this study retained 9 residents out of 23 for at least 9 sessions over a 12-week period with an average of 11 residents participating per class. Since the TC class was open to all, some of the attendants came to only one session just to find that TC was not for them. However, one of the residents who had never participated in any of the other exercise classes at the facility attended all 12 TC classes. According to the Community Life Director, one of the reasons for the high interest in TC program could be the fact that the existing exercise program had been in existence for nearly five years and the residents were eager to learn some new movements. Anecdotally, one of the participants took great pride in teaching TC to several police officers who visited seniors at the project site.

This small quality improvement study demonstrated that building a TC program was feasible and safe, providing additional support and evidence for offering TC programs in other senior living communities. Although sustainability was not a stated goal and was beyond the scope of this project, both feasibility and sustainability are considered measures of successful implementation of a quality improvement project. All of the positive outcomes generated through this project have no real value if the program cannot be readily replicated and sustained in the facility. Fortunately, a recent update received from the assisted living facility indicated the TC program has been thriving for the past three years after the implementation. Staff from the assisted living facility reported that the supporting materials provided at the end of the program were very helpful in supporting the program, and staff noted TC practice not only improved residents’ physical function but also helped reduce stress and anxiety especially during the Covid-19 pandemic. Taken together, the results of our preliminary analysis provide evidence to support the feasibility and acceptability of the TC program in an assisted living facility.

This project suggested that TC may have a potential to improve functional mobility and reduce the fear of falling which in turn may reduce fall risks as evidenced by a clinically important change in TUG times and a decreasing trend in FES-I score observed from pre- to post-intervention. The TUG test is often used to predict fall risk and is highly correlated with balance confidence [30]. The results from this project are consistent with the findings from other studies [14,18,31]. Fear of falling is another indicator for fall risk, and 20% improvement in FES-I score in this program suggested a potential to increase confidence in carrying out daily activities among the participants. This result aligns well with the findings from the recent RCT study indicating practicing TC at a minimum of four weeks could help reduce fear of falling [32]. No clinically important changes in QoL were observed in this project, which is also consistent with a meta-analysis that showed no significant association between exercise and the measures of QoL [33]. However, the participants had already self-reported high level of QoL at pre-intervention. There might be some possibility that older adults who would choose to try a new practice like TC already have a positive outlook on life and thus perceive their quality of life to be high. QoL is a long-term trait and is generally known to be a hard factor to alter, more so in such a short term. In addition to needing a longer intervention period, larger sample size and a wider diversity of participants might have yielded a clinically important change in QoL.

This project represents a nurse-led TC program in an assisted living facility. The nurse was a trained novice TC instructor, supporting the evidence of effective implementation of a TC program by novice instructors [19]. The project leader and TC instructor used a theoretical framework to guide the project implementation and intentionally build a cohesive TC community by providing a supportive and encouraging environment. The instructor offered modifications such as seated or chair assisted movements, based on the learning capacity of the participants to increase their self-efficacy in learning. The instructor intended to create a fun and supportive environment, offering verbal praise and encouragement, as well as greetings and check-in with residents before and after class. Snacks were provided at some of the sessions, and small holiday gifts were given to residents at the end of the project. The actual TC class participation exceeded the expected attendance rate and the post-intervention survey showed 100% satisfaction among the participants. One can surmise that instructor attention to participant needs impacted the relative success of this program and should be considered in future program planning.

There are several project limitations. This TC program was offered with less frequency and shorter class time than TC studies cited in the literature; most TC protocols consisted of 12 weeks or longer intervention with a 60-min TC training 1–3 times per week [16,31]. However, systematic literature review suggested that conducting exercise classes in small groups with less frequency and duration may enhance the adherence rate since a higher dropout rate is a concern in TC program implementation [29]. In addition, a recent RCT indicated there was no significant increase in benefits for groups who received TC twice weekly compared with once weekly [12]. Furthermore, this facility offered exercise activities and a wellness exercise program (30 min per day), which informed the decision to offer a 45-min weekly TC class.

Second, due to the lack of randomization and control group as well as the fact that the project was not powered to assess effectiveness, no cause-effect conclusion could be drawn. Third, competing events such as other exercise classes that occurred simultaneously in the facility may be confounding factors affecting the results. Controlling for these factors was not feasible in this project. Another consideration is that the facility was relocating the dining space during the implementation period and altered the mealtime, which significantly reduced the number of participants in the exercise classes, and this could have affected TC class attendance. In addition, there was lower participation during the holiday season as many residents spent the holidays with family. Self-reported satisfaction may be affected by reporting and desirability bias. Implementation of the TC program in one assisted living facility and a convenience sample used limit the generalizability of the results.

## 5. Health Care Implications

Patient safety is always the first priority for healthcare providers. Identifying fall risks and preventing falls are key components of caring for the aging population. Healthcare providers should not only receive education on the traditional approach of fall prevention (e.g., risk assessment, hourly safety rounds, etc.), but they should also learn alternative activities to reduce risk of falling in older adults, such as an evidence-based TC practice. Prelicensure and graduate gerontological curriculum could include selected elements of TC programs to increase the knowledge and skills related to TC practice. From our experience, participants feel encouraged and more confident if their healthcare providers support and reinforce the benefits of practicing TC. In addition, healthcare providers can learn to be effective in teaching TC and assume leadership in the development and implementation of TC programs for older adults.

## 6. Conclusions

As the generation of baby boomers age and lifespan continues to increase, the aging population is projected to increase. It is plausible, therefore, that more senior residents will occupy assisted living facilities. Healthcare providers in these senior living facilities play a crucial role in providing leadership in geriatric care and introducing appropriate evidence-based interventions, such as an exercise intervention like TC. This project demonstrated that establishing a TC program by a novice TC instructor in an assisted living facility was feasible; the TC program was safe and was well-received by older adults in the community setting as well as by health providers. The program might have a potential to improve functional mobility and reduce fear of falling, which in turn might reduce risk of falls. In addition, staff in senior living communities could be trained and certified as TC instructors to assist with implementing the evidence-based TC program and to sustain the program in the long run. Future research is needed to assess the effectiveness of TC programs to reduce risk of falls among older adults in community settings utilizing an appropriately powered randomized-controlled trial design and a longer intervention period.

## Figures and Tables

**Table 1:** FES-I, TUG, and Older People’s Quality of Life (OPQOL) Results.

Measures	FES-I		TUG		OPQOL	
	Pre	Post	Pre	Post	Pre	Post
Mean (SD)	15(6.2)	12(4.3)	22.4(7.62)	17.6(7.14)	54(7.2)	55(7.3)
Range	7–26	7–23	12.8–38.2	10.7–32.3	43–64	40–64
Mean Change (absolute, units)	−3		−5		+1	
Mean Change (relative, %)	−20%		−21%		+1.9%	

FES-I: Falls Efficacy Scale-International; TUG: Timed Up and Go.

**Table 2:** Single Item-global quality of life (QoL) Score Summary Statistics.

	Pre	Post
QOL Score, *n* (%)	(*n* = 11)	(*n* = 11)
1—Very bad	0	0
2—Bad	0	0
3—Alright	3(27%)	1(10%)
4—Good	6(55%)	5(45%)
5—Very Good	2(18%)	5(45%)

## Data Availability

The data presented in this study are available on request from the corresponding author. The data are not publicly available due to the small size of the program.

## Appendix A. Tai Chi Program Satisfaction Survey

The purpose of this survey is to receive feedback on the weekly Tai Chi program. Your participation and support in facilitating this project are greatly appreciated.

1. How many Tai Chi classes did you attend? ________________ (1–12)
2. What prevented you from attending some of the Tai Chi classes?
☐ Sick (e.g., cold, flu, other health problems)
☐ Hospitalization
☐ Conflict with schedule (e.g.,: outings, doctor’s appointment, visitors)
☐ Limited mobility
☐ Others

3. I enjoyed the weekly Tai Chi class very much.
☐ Strongly agree
☐ Agree
☐ Disagree
☐ Strongly disagree
4. What was your favorite part of the Tai Chi classes?
☐ Learning Tai Chi movements
☐ Breathing exercise
☐ Using Imagery (e.g.,: imagine you are a tree rooting down to the earth, imagine you are a flying goose opening up your wings)
☐ Opportunity to engage in teaching (e.g., counting, naming a few tai chi movements you’d like to do)
☐Social interaction
Other_________________________________________________________
5. What was your least favorite part of the Tai Chi classes?
☐ Learning Tai Chi movements
☐ Breathing exercise
☐ Using Imagery (e.g.,: imagine you are a tree rooting down to the earth, imagine you are a flying goose opening up your wings)
☐ Opportunity to engage in teaching (e.g., counting, naming a few tai chi movements you’d like to do)
☐ Social interaction
Other_________________________________________________________
6. The weekly Tai Chi was very helpful to my health and wellbeing.
☐ Strongly agree
☐ Agree
☐ Disagree
☐ Strongly disagree
7. How did the Tai Chi program benefit you? (select all that apply)
☐ Improve strength
☐ Improve flexibility
☐ Improve balance
☐ Improve mood
☐ Improve energy
☐ Reduce pain
☐ Enhance relaxation/reduce stress
☐ Add fun and joy to life
Other__________________________________________
8. Would you recommend this class to your friends and neighbors?
☐ Yes
☐ No
☐ Maybe
9. Would you like this Tai Chi program to continue?
☐ Yes
☐ No If no, why_______________________
10. What other comments do you have about the Tai Chi class?
